# ViralFP: A Web Application of Viral Fusion Proteins

**DOI:** 10.3389/fmedt.2021.722392

**Published:** 2021-08-23

**Authors:** Pedro Moreira, Ana Marta Sequeira, Sara Pereira, Rúben Rodrigues, Miguel Rocha, Diana Lousa

**Affiliations:** ^1^Centro de Engenharia Biológica, Escola de Engenharia da Universidade do Minho, Braga, Portugal; ^2^ITQB NOVA, Instituto de Tecnologia Química e Biológica António Xavier, Universidade Nova de Lisboa, Oeiras, Portugal

**Keywords:** fusion proteins, fusion peptides, database, web application, machine learning

## Abstract

Viral fusion proteins are attached to the membrane of enveloped viruses (a group that includes Coronaviruses, Dengue, HIV and Influenza) and catalyze fusion between the viral and host membranes, enabling the virus to insert its genetic material into the host cell. Given the importance of these biomolecules, this work presents a centralized database containing the most relevant information on viral fusion proteins, available through a free-to-use web server accessible through the URL https://viralfp.bio.di.uminho.pt/. This web application contains several bioinformatic tools, such as Clustal sequence alignment and Weblogo, including as well a machine learning-based tool capable of predicting the location of fusion peptides (the component of fusion proteins that inserts into the host's cell membrane) within the fusion protein sequence. Given the crucial role of these proteins in viral infection, their importance as natural targets of our immune system and their potential as therapeutic targets, this web application aims to foster our ability to fight pathogenic viruses.

## 1. Introduction

Enveloped viruses, a group of viruses that includes SARS-CoV-2, Dengue, Influenza and HIV, are characterized by having an exterior lipid envelope that contains at least one type of glycoprotein on its surface (1, Preface I) (2, 3). These proteins play an essential role during the virus entry into the host cell, enabling the binding of the virus to receptors on the host cell and catalyzing the fusion between the viral envelope and the host cell membrane.

In some viruses, a single protein can induce the binding to the host receptor and catalyze the fusion reaction, whereas other viruses require multiple proteins for these tasks. Viral fusion proteins (VFP) are key players in a process called membrane fusion, through which the virus inserts its genetic material into the host cell, enabling it to replicate (4, 5).

During membrane fusion, the viral envelope and the host cellular membrane are brought into close contact in order to merge into a single membrane (6, 7). According to the most widely accepted membrane fusion model, this process is initiated when the VFP binds to the host receptor. Triggered by an external stimulus, the protein becomes extended and inserts its fusion peptide (FPep) into the host membrane. The VFP folds back, which leads to the approximation of the two membranes. After this step, the mechanism passes through a hemifusion intermediate, which encompasses the fusion of the outer leflets of the membranes without the contact of the inner leaflets. This hemifused step forms a stalk-like structure, diminishing the contact area to a minimum, lowering the free energy barrier required to overcome hydration repulsion. Finally, fusion of the unattached inner leaflets occurs, which starts to form a hydrophilic pore that will eventually enlarge, connecting the 2 initially separated intermembrane volumes (4, 5, 8–10).

VFPs can assume multiple possible forms depending on their corresponding virus. Currently, the consensus is that VFPs can be grouped into 3 classes: I, II, and III, each with their own characteristics, such as their prefusion, pre-hairpin and post-fusion structures, orientation within virus membrane, types of possible triggering mechanisms and location of their FPep (4, 5, 7, 11).

The FPep is a conserved sequence within VFPs of the same viral family that can insert into the host membrane, allowing the fusion of the viral envelope with the host cell membrane. This peptide can promote the reaction of fusion of membrane vesicles by itself, even if it is detached from the rest of the VFP (4, 11). All FPeps share common characteristics, which are determinant for their function: they are hydrophobic, rich in Gly and Ala residues, contain aromatic residues and are usually conserved within a species (mutations frequently lead to a loss of function) (4, 11). Apart from these general characteristics, FPeps from virus belonging to different families can be quite diverse (4). Some FPeps (e.g., Influenza and HIV) are located at the N-terminal tip of the fusion protein, whereas the peptides of other viruses (e.g., Dengue and Ebola) are internal fusion loops (4). The peptides from different families are also quite different at the sequence level and structure levels. The influenza FP is helical in lipidic environments (12, 13), whereas the HIV FP tends to adopt β-sheet structures (14), although it can become helical depending on membrane composition (15). Other FPeps, such as the one from Dengue virus, which only has 14 residues, do not have a defined secondary structure (16). It is not clear how peptides with such distinct characteristics play a common role in membrane fusion.

The relevance of VFP and their FPep for membrane fusion highlights them as potential therapeutic targets (4, 7). As an example, the therapeutic application of VFPs as a target can be found in the defining pandemic of 2020: COVID-19. Being an enveloped virus, one of the key therapeutic targets of SARS-CoV-2 is its VFP, the spike (S) protein (17). The most promising and effective vaccines under development or already administrated for this virus are based on the S protein's mRNA. This group of vaccines include the Moderna's mRNA-1273 (18) and the Pfizer-BioNTech's BNT162b2 mRNA (19). In both, the mRNA molecule is inserted into a lipid nanocarrier that stabilizes the mRNA and delivers it into human cells. The mRNA once inside a cell is translated to produce the S protein in its prefusion conformation. The presence of those proteins in the human cells induces an immune response on the organism, which will produce antibodies that can target those proteins. For several other enveloped viruses there are known antibodies that target that region, like in cases of Dengue (20), Zika (21), HIV (22), and Influenza (23).

The known information for a given VFP can be found in biological databases, like UniProt (24), NCBI Protein (25) and PDB (26). However, a VFP oriented repository that contains VFP related information does not exist, forcing researchers to search several databases when they need to have comprehensive characterization of one or several of these proteins.

To better understand how VFPs and their components are characterized, some bioinformatics tools can be used, like sequence alignment [e.g., BLAST (27), Clustal (28), and HMMER (29)], that allow researchers to discover a protein's conserved residues, as well as other pieces of information regarding their features. These analyses can provide valuable insights about how VFPs operate.

Researchers have also been using machine learning (ML) to study VFPs and their inhibitors. Several promising tools have been developed using ML models trained using VFP and their features, allowing the prediction of FPeps location within VFP sequences (30, 31) and the prediction of inhibitors for different VFPs to potentially be used in therapies of some diseases (32).

In this context, the main objective of the current work is to develop a CRUD (Create, Read, Update and Delete) web application, named ViralFP, that is capable of storing, retrieving, displaying, and analysing the gathered data on enveloped viruses [through information gathered from NCBI Taxonomy (33) entries] and their VFPs, as well as to enable the data navigation through a user-friendly interface. The web application is freely available and aims to be useful for all elements of the scientific community who are working with VFPs and FPeps. The underlying data are stored in a relational database, which contains relevant information regarding over 500 VFPs. Manual curation of the database information was performed by reviewing the original protein and taxonomy repositories' data entries, as well researching through scientific publications.The back-end component of the application is built using Django and Django REST API library, whilst its front-end is built using Angular technology, a Typescript framework to build web applications. This web application also includes a set of selected bioinformatics tools, such as sequence alignment tools (like BLAST and Clustal) and ML models capable to detect FPeps within the VFP sequence.

## 2. Materials and Methods

**Figure 1:**
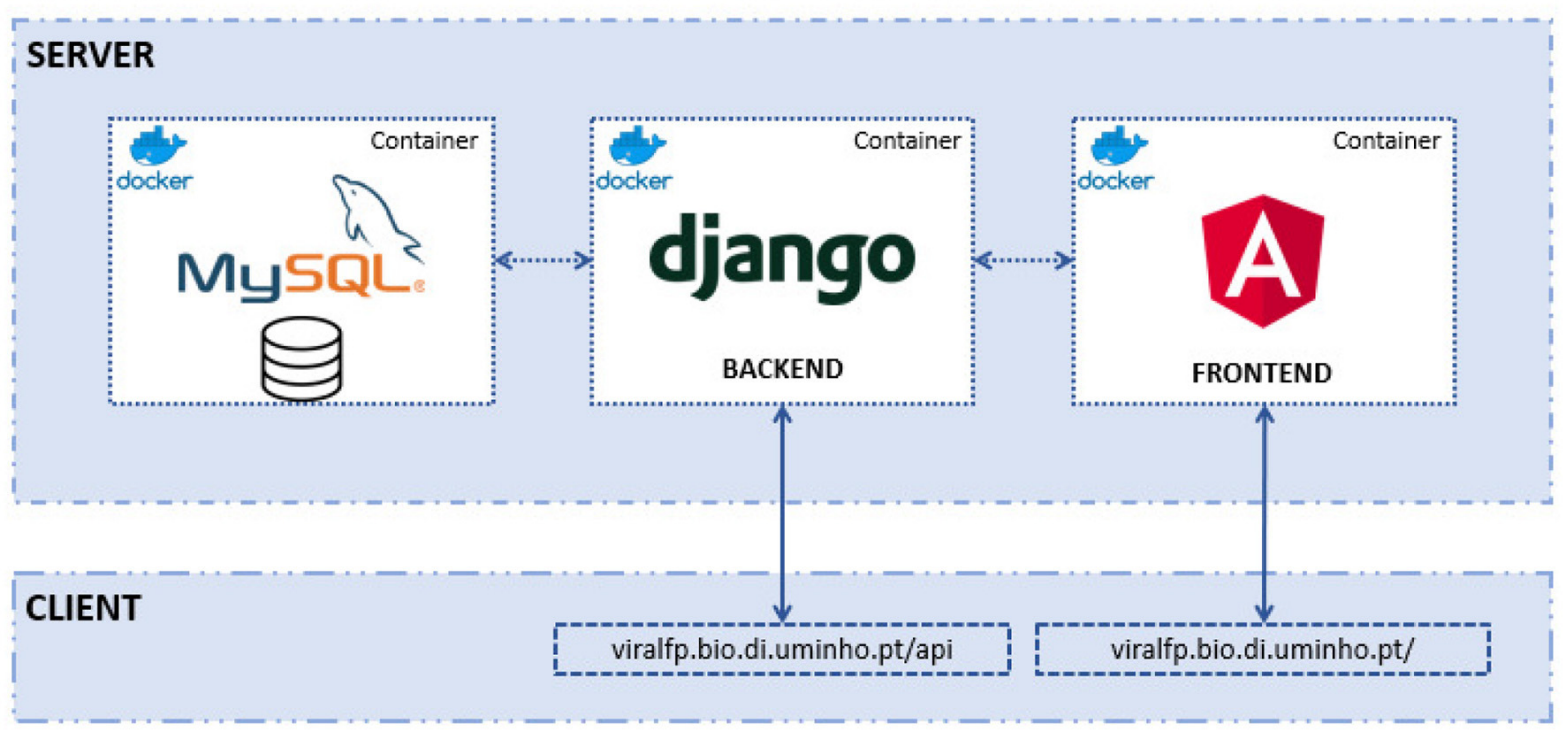
Structure of the app: within the deployment server there are the 3 Docker containers: the MySQL, with the relational database; the Django (back-end) and the Angular (front-end). The latter two can be accessed by a web client (e.g., browser or any application that a consumes RESTfull API).

The structure of the ViralFP application can be seen in the Figure 1. There are three distinct components, each with its own Docker container:

- The MySQL database-stores all the data that the server requires.
- Django back-end, with Django REST framework–enables the access and management of the database, besides containing several data views that will be called by the front-end.
- Angular front-end-the application designed to be used by the end user that displays the database information and allows the execution of several tools.

In the next sections, a deeper explanation for each of those components will be made.

### 2.1. Database

**Figure 2:**
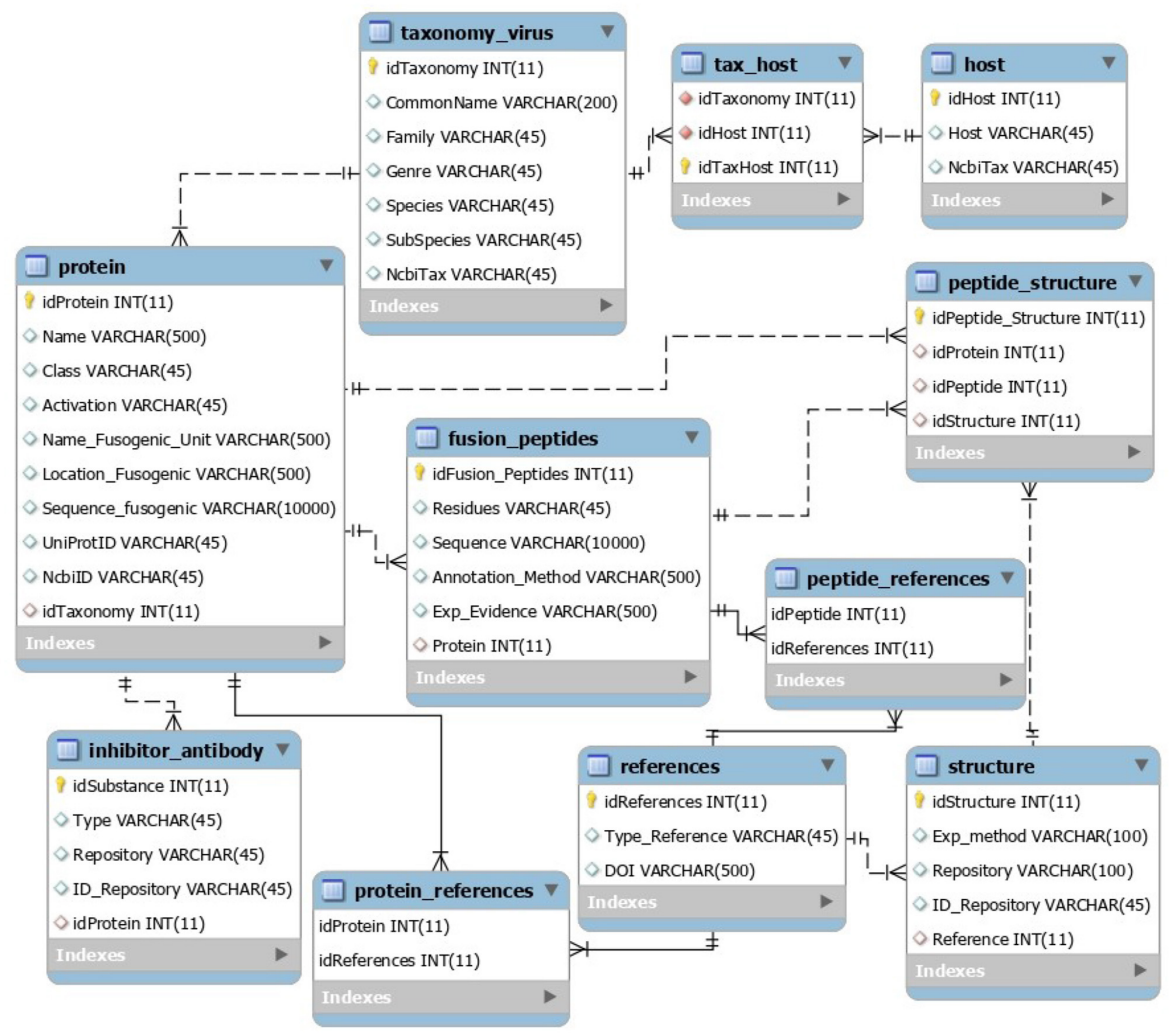
Structure of the relational database, showing its entities and relationships.

The VFP/FPep data present in the MySQL database was retrieved from UniProt, NCBI Protein and PDB entries, whilst the taxonomy information was originally from NCBI Taxonomy. Its relational structure, names of the tables and attributes, as well as the type of data stored in each attribute can be seen on the scheme present in Figure 2.

The core table of this database is called Protein and contains the information regarding each VFP's name, class, type of activation, fusogenic peptide description (its name, location within the protein and sequence), as well as having the UniProt and NCBI Protein entries for the protein.

Another important table is the Fusion Peptide, which describes the location of this peptide within the VFP sequence (attribute Residues), sequence and some attributes regarding annotation method and experimental evidence.

The last key table is the Taxonomy, which has all the relevant taxonomy of the virus to which the protein belongs to (family, genus, species, and subspecies/strain), as well as having the virus NCBI Taxonomy entry.

Additional tables within the database include a table with the possible structures of the VFP (through PDB entries); a table for the virus' hosts, which has the common name and NCBI Taxonomy of each virus host organism; an inhibitor/antibody table, that contains information regarding those types of protein that are associated with a particular VFP, and, finally, tables for additional bibliographical references.

The data present in the database was manually curated by verifying the original repositories of the data entries. Finally, the inhibitors and antibodies entries for each VFP were filled using *BioPython* (34) for the Pubmed automated searches, and *pypdb* (35) for the PDB searches.

### 2.2. Back-End

The main purpose of the back-end is to enable the access to the data from the MySQL database. This application component converts data from SQL queries into JSON objects. Those objects can be viewed and managed by a web client, which in this case implements the front-end. The back-end has a RESTful API which enables the submission or modification of data stored in the MySQL database.

One view for each database table was built with the same name of the respective table. Some of those views use serializers that retrieve data from multiple tables related between themselves by foreign keys. Whenever a query request is made, the returned data will be returned as response (also in a JSON format). All these pages will allow to receive requests to insert, update or delete data entries. Besides that, all of those will contain features to allow to filter and/or search data, besides containing pagination methods.

Since bioinformatics packages are commonly written in Python, those tools are integrated within this application layer, that later will be accessed by the front-end by requests.

Clustal Omega analysis was wrapped in our application using a Clustal console (36). This view will receive the sequences and the additional parameters as part of the request. As result, it performs a Clustal alignment which provides the sequence alignment output as a text response.

To build Weblogos, the packages *weblogo*^1^ and *logomaker* (37) are used.

The ML view allows to perform the FPep prediction for a given sequence using models trained with the in-house *Propythia* package (38). This package enables the development of ML and deep learning models for classification of peptides. It allows to obtain protein descriptors from their sequences such as physicochemical features, and includes methods for data preprocessing, feature selection, dimensionality reduction, clustering and pipelines to train, evaluate and optimize ML and deep learning models (38). The models used in this application were trained from a dataset generated by extracting the physicochemical features using the Descriptor module of Propythia from a dataset where the negative instances are composed of protein sequences from transmembrane domains and random sequences extracted from VFP sequences after excluding the FPep. The group of descriptors that the Descriptors module provide include physicochemical features calculated from the peptide's sequence (e.g., length, charge, charge density, number of C, H, N, O, and S atoms within the amino acids, molecular weight, gravy, aromacity, isoelectric point, instability index, secondary structure, molar extinction coefficient, flexibility, aliphatic index, Boman index and hydrophobic index); its amino acid and pseudo-amino acid compositions (including dipeptide and tripeptide), CTD (Composition, Transition and Distribution) information, Conjoint Triad and sequence order, as well additional base class peptide features. These descriptors are obtained from functions available in the packages *PyDPI* (39), *Biopython* (34), *modlAMP* (40), and *pfeature* (41). Calculated features went through a wrapper feature selection process using a Support Vector Machine (SVM) model, which had the best performance in this dataset. From these features, the ones that were considered as the most relevant include tripeptide and dipeptide composition, secondary structure, autocorrelation and CTD descriptors. Finally, ML models were trained with the Propythia's *train*_*best*_*model* function from the shallow machine learning class. Those models are stored in Pickle files^2^.

If a certain model is selected, the file containing that model will be loaded and used in an instance of the *Propythia*'s ML class. This ML view also requires the selection of the window size and a size for the gap. After defining those parameters, the *predicted*_*window* function of the Propythia is executed to perform the likelihood prediction of the subpeptide present in a given window to be a FPeps. Those probabilities are returned in a JSON object by the view.

### 2.3. Front-End

The front-end was built using a framework called Nebular^3^, which is an open-source Angular User Interface Library that consists in a pre-built modular, customizable and configurable Angular tool. This framework enables the creation of Angular-based web applications and speeds up the development process of a front-end by providing several useful user-friendly and visually appealing web components, like tables and card formats.

#### 2.3.1. Tables' Pages

The information of the database is found on the “Tables & Data” option of the front-end menu. This menu option contains a list of links for the Taxonomy, Fusion Protein and Fusion Peptides tables. All tables are organized in a Tree Grid format: each data entry will correspond to a table line; upon clicking on one row, its related data, which in this app corresponds to links to other tables connected by foreign keys, will appear below.

#### 2.3.2. Sequence Alignment Pages

The application's tools can be grouped together in two categories: sequence alignment and sequence prediction. This section will cover the first group, while the next section handles the latter.

In the Sequence Alignment tools, a page that enables the submission of sequences to the NCBI BLAST was added. This page encompasses parameters that allow the selection of the type of BLAST and the database which BLAST will run against (27). Similarly, the HMMER page allows the selection of a sequence and the database to perform the alignment in the EMBL-EBI portal, showing the results in their web portal (42).

The Weblogo page contains two possible ways to build sequence logos. The first uses the Weblogo 3 portal^4^ (43), in which the page will ask a minimum of three sequences, which must be in a FASTA format. Those sequences are submitted to the back-end to perform a Clustal Omega alignment and from that the web portal builds a weblogo provided in PNG or raw data formats. The second option to generate weblogos consists in the usage of the integrated sequence logo generator of the back-end. The PNG output will appear in the same page, on which the user can select the type of coloring provided by the *logomaker* package, as well as the number of stacks per line; the text output will appear in a pop-up page.

The Clustal page alignment will be run in the back-end, in a command line. The user can select parameters such as the format of output. Upon submitting the request, one pop-up will open in the browser to display the alignment in the required format; a second one can appear if the guide tree data was requested.

#### 2.3.3. Sequence Prediction Pages

The web application contains two tools relative to sequence prediction: epitope prediction and FPep prediction.

Regarding the epitope prediction (epitopes are binding sites that antibody molecules can attach to) the site will access to the IEDB portal (44) to obtain an antibody epitope prediction. The page will send a request with the sequence, selected analysis method and window size to the back-end that will redirect to the IEDB API. The results will be displayed in a Smart Table format. Both Bepipred methods' results will appear into 2 different result tables, the rest of them just require one (45).

Regarding ML-based prediction of the location of FPeps within the VFP, this page allows to define a VFP target sequence, as well as specifying which model to use, from the set of available models trained in Propythia: Support Vector Machine, Random Forest, Gradient Boosting, K-Nearest Neighbors, Linear Regression, Gaussian Nave Bayes, and Artificial Neural Network models. Finally, the user can select the window and gap sizes. The page can display those predictions in two types of output: a table which contains every possible peptide probability of being a FPep; and a graphical interface that, for each amino acid, displays a color that represents the maximum probability of all peptides that contain that particular character of being a FPep. Moreover, for each amino acid, a popup box will show up, informing the user about the position within the VFP sequence and the probability value of belonging to a FPep. These results can be downloaded into a text file.

## 3. Results

There are two key components of the front-end that can require an explanation of their elements: the tables and the tools.

**Figure 3:**
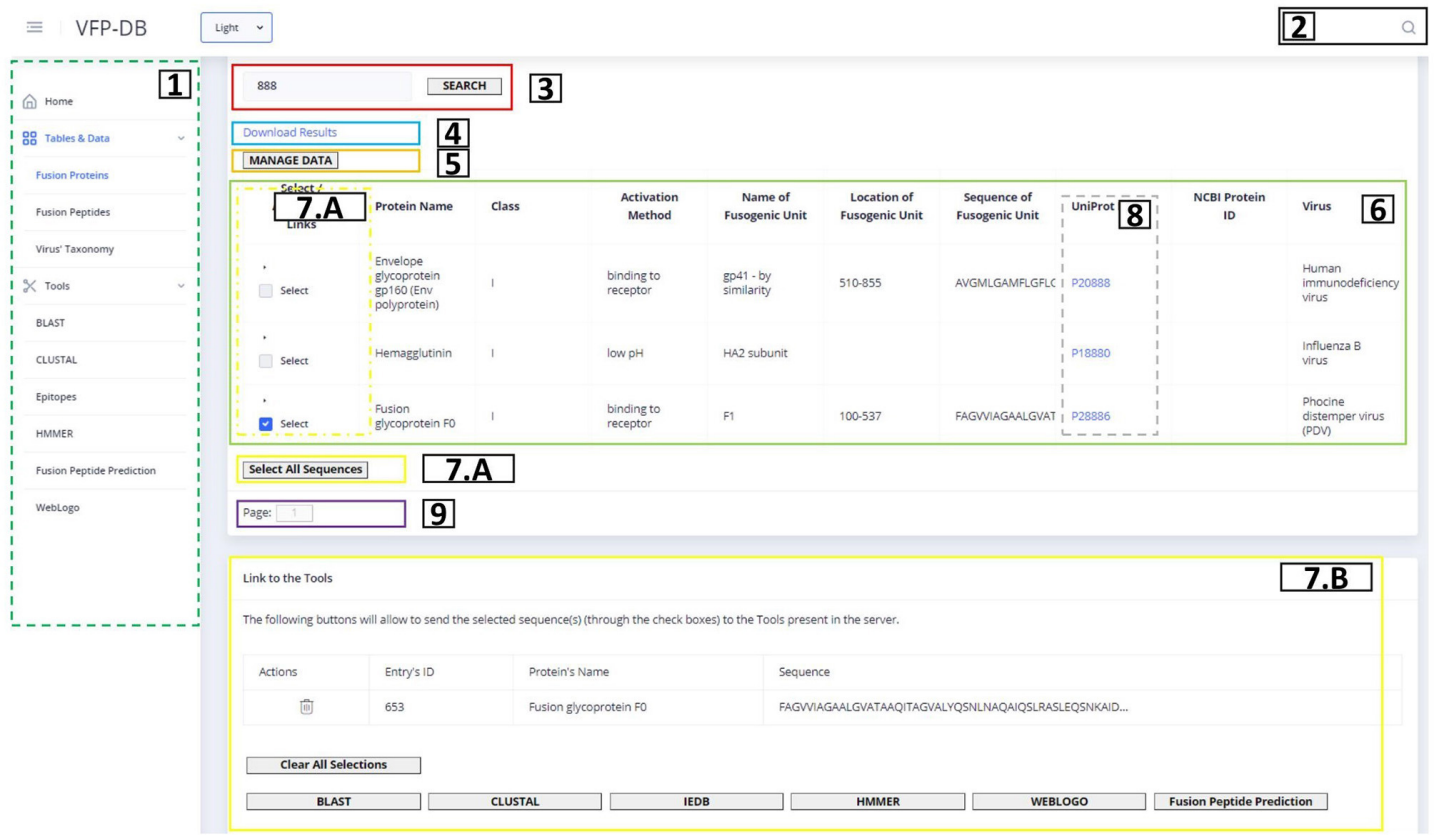
Fusion protein page scheme.

Figure 3 shows the representation of the Fusion Protein page. This image contains an example of a table component and will be used to explain all table elements present in our web application. The list above corresponds to the description of the table functionalities from the left to the right and top to down:

- On the dashed green rectangle on the left (number 1), there is the side menu of the application, where the user can access the three main data tables, as well as the bioinformatics tools present in the server.
- On the top right, in the black box (number 2), the user can find the link to the global search tool; upon entering a search term, a pop-up window will be displayed if the main pages contain results.
- The red rectangle (number 3) contains the search methods previously described, that will determine what is shown in the table; the search box contains an auto-complete system with term suggestions that is executed after the input of 3 characters.
- The blue rectangle (number 4) allows to download the query result into a CSV file.
- The orange rectangle (number 5) redirects to the back-end's administrator page.
- The green rectangle (number 6) is the table with results from the query request determined by the search term and the current page; within each line, there are links to related data (if available).
- The yellow rectangles (numbers 7.A and 7.B) show the selection of sequences that will be provided to the web application tools, chosen in the checkboxes on the yellow dashed rectangle, or through the “Select All Sequences” button; upon choosing at least one sequence, the rectangle visible at 7.B will show up, allowing the user to delete some (in the table) or all (in the “Clear All Sequences” option) of the selected sequences and redirect the chosen sequences to the Tools, accessible by the buttons on the bottom of the page.
- Some columns like the one inside the dashed gray rectangle (number 8) will contain links to redirect to the original source of the data; the links can be UniProt, NCBI Protein, PDB or NCBI Taxonomy data entries.
- The purple box buttons (number 9) correspond to the pagination methods that will also determine the content of the table.

**Figure 4:**
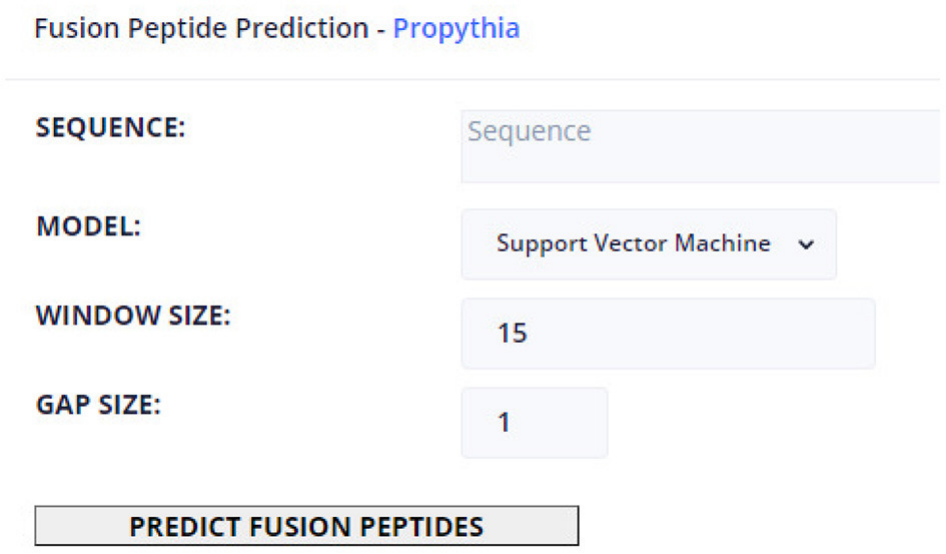
Machine learning page.

**Figure 5:**
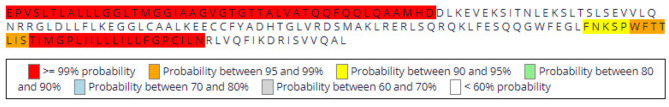
Example of a machine learning prediction of the fusion peptide location.

Figure 4 shows an example of how all tool pages are structured. Regarding the available tools, they contain a text box to insert the sequence(s) input, which can be auto-filled if protein sequences were selected in the Fusion Protein page: some tools (e.g., the multiple alignment tools) require a minimum of 3 protein sequences in a FASTA format; however, the most basic input of the text box is a single protein sequence. To instruct the user about the type of input that each tool requires, the text boxes contain placeholders with a small description of expected input. After the text box is filled, the user can set custom values for each of the rest of the parameters. The results of the tools can appear in new tabs with text results (e.g., using the Clustal or Weblogo), or to access other web portals (e.g., BLAST or HMMER). The results can also appear within the same page, like in the case of ML prediction (as shown in Figure 5 and the image result of Weblogo built from the back-end's functions.

### 3.1. Use Case-Analysis of the *Retroviridae* Conservation

**Figure 6:**
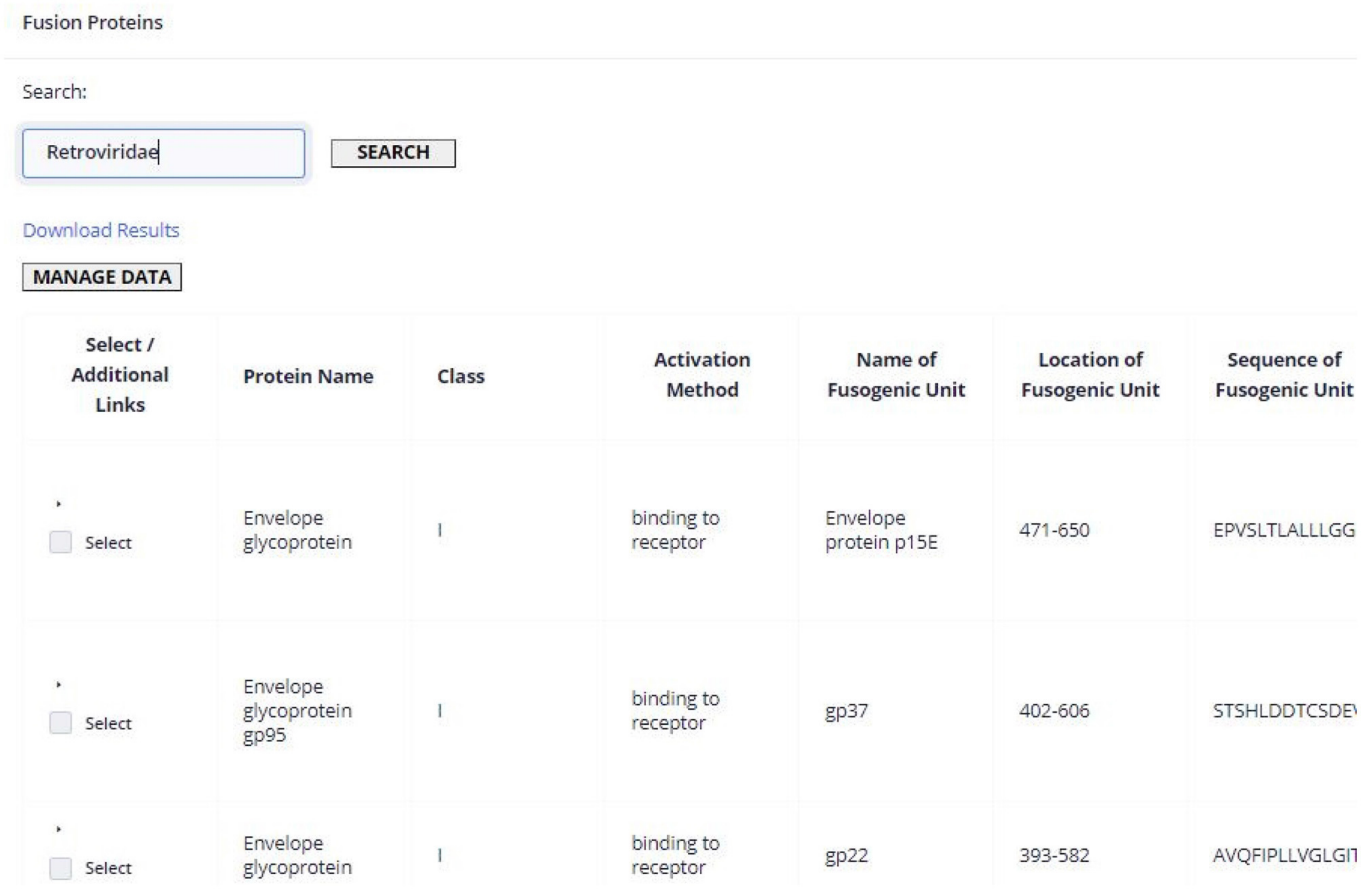
Hits retrieved upon querying the Fusion Protein page using the search term “*Retroviridae*.” For the sake of simplicity only the first hits are shown.

To illustrate the application of ViralFP, we describe the case of a researcher that aims to obtain a general perspective of the conservation of the VFP of a given viral family, such as *Retroviridae*, to determine which regions could be interesting universal therapeutic targets. This can be done by accessing the Fusion Protein page, searching the “*Retroviridae*” term (Figure 6), selecting all the sequences, and sending them to the Weblogo tool page through the corresponding option.

**Figure 7:**
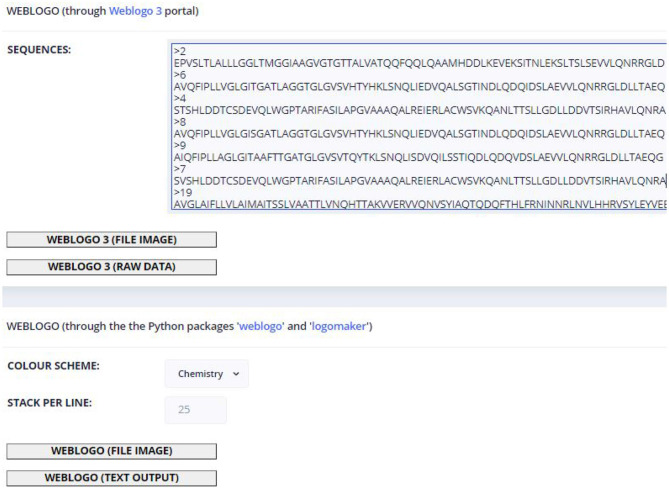
View of the weblogo page with the *Retroviridae* fusion protein sequences provided as input.

The sequences sent are already in the required FASTA format. If the user wants to obtain the graphical option of the weblogo, he/she can select the appropriate coloring to explore the conservation (in the case of Figure 7 the selected coloring is based on the amino acid physical-chemical properties) and the number of characters per line (default: 25).

**Figure 8:**
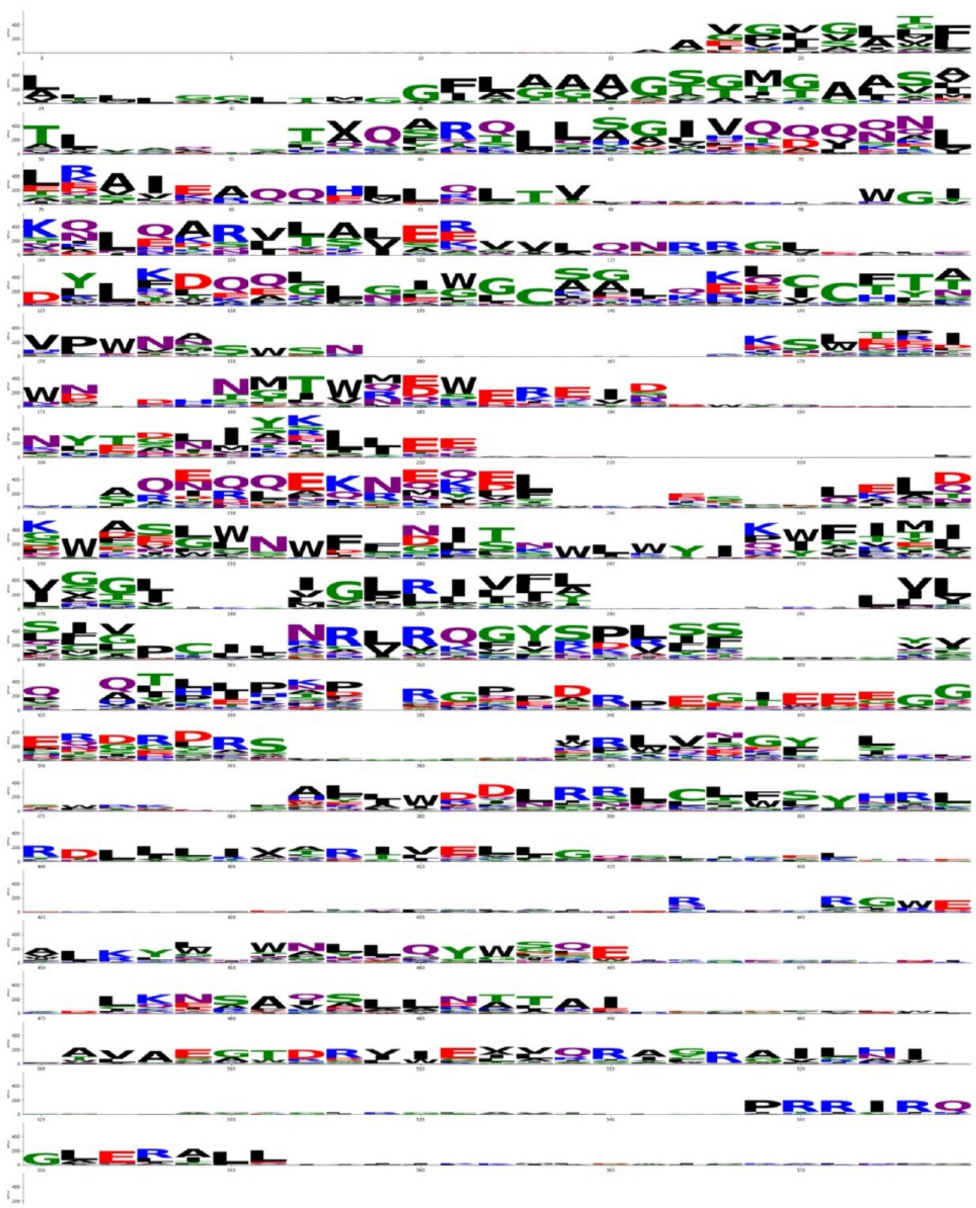
Weblogo analysis of the fusion proteins from the *Retroviridae* family.

After running the analysis, the output is shown below (Figure 8). Using this representation, the regions with high conservation can be easily located, since they correspond to positions with larger stacks. In those, the conserved region found around positions 15 (first line) through 60 (third line) corresponds to the FPep, a very conserved region.

## 4. Discussion

This work provides a useful resource for the scientific community working with viruses and their proteins, since it presents a tool that can be used by any researcher interested in obtaining several types of information regarding VFPs.

Currently, the application implements several useful functionalities: it provides an easy access to the MySQL database containing VFP information through a user-friendly web interface. The proposed bioinformatics tools are implemented in the application, through web interfaces. ML models capable of predicting FPeps within the VFP sequence are also available. Finally, a full manual curation of the database information was performed.

Future tasks within the website will include improving the tool by improving current features and adding new ones, including the possibility to define more parameters within the application's tools, improving the initial loading time of the website and the app's aesthetic appearance.

Alongside these tasks, to improve the utility of this application for the scientific community, we will try implement tools capable of predicting the most conserved regions within proteins, which will allow to complement the ML output, due to FPeps tending to be conserved regions. ML models will be improved as new data regarding FPeps is discovered and added to the database, allowing us to update and expand its training dataset.

Regarding the database's information, it is imperative to add all the relevant new entries published after the submission of this manuscript, hopefully by implementing methodologies capable of performing this type of search automatically and periodically. We are going to add methods that allow users to insert new data, as well as update some inconsistencies that they might find in the application. We aim to add information regarding receptors of VFPs, as well as each VFP's known cleavage sites. Finally, it is important to redo the automated searches of known inhibitors/antibodies within Pubmed and PDB in a way that can take into consideration the virus' strain.

## Data Availability Statement

Publicly available datasets were analyzed in this study. This data can be found here: https://github.com/pdMM11/Dockers/tree/master/Django_/crmapp/ml_models/DATASETS.

## Author Contributions

DL, MR, and PM conceptualized the web application and wrote the manuscript. PM was the main developer of the web application, by implementing all the different components that allow the functioning of the front-end, back-end and integration of bioinformatics tools, procedures described in the Materials and Methods chapter. SP was responsible for gathering the information present in the protein repositories that later was inserted into the web application database. RR coordinated the development of the web application and deployed it into the server. AS developed *Propythia* and the ML pipelines used to train the models present in the application. DL, MR, and RR supervised the work. All authors revised the manuscript.

## Conflict of Interest

The authors declare that the research was conducted in the absence of any commercial or financial relationships that could be construed as a potential conflict of interest.

## Publisher's Note

All claims expressed in this article are solely those of the authors and do not necessarily represent those of their affiliated organizations, or those of the publisher, the editors and the reviewers. Any product that may be evaluated in this article, or claim that may be made by its manufacturer, is not guaranteed or endorsed by the publisher.

## Footnotes

^1^ https://weblogo.readthedocs.io/

^2^The datasets, as well a small tutorial on how the models were trained, can be seen in https://github.com/pdMM11/Dockers/tree/master/Django_/crmapp/ml_models/DATASETS

^3^ https://akveo.github.io/nebular/

^4^ http://weblogo.threeplusone.com/create.cgi

## Abbreviations

FPep: Fusion Peptide
ML: Machine Learning
VFP: Viral Fusion Protein.
